# Neuroprotection in Stroke: Past, Present, and Future

**DOI:** 10.1155/2014/515716

**Published:** 2014-01-21

**Authors:** Arshad Majid

**Affiliations:** ^1^Department of Neuroscience, Sheffield Institute for Translational Neuroscience (SITraN), University of Sheffield, 385A Glossop Road, Sheffield S10 2HQ, UK; ^2^Department of Neurology and Manchester Academic Health Sciences Centre, Salford Royal Hospital, Stott Lane, Salford M6 8HD, UK

## Abstract

Stroke is a devastating medical condition, killing millions of people each year and causing serious injury to many more. Despite advances in treatment, there is still little that can be done to prevent stroke-related brain damage. The concept of neuroprotection is a source of considerable interest in the search for novel therapies that have the potential to preserve brain tissue and improve overall outcome. Key points of intervention have been identified in many of the processes that are the source of damage to the brain after stroke, and numerous treatment strategies designed to exploit them have been developed. In this review, potential targets of neuroprotection in stroke are discussed, as well as the various treatments that have been targeted against them. In addition, a summary of recent progress in clinical trials of neuroprotective agents in stroke is provided.

## 1. Introduction

Stroke is one of the leading causes of death and disability worldwide. Despite decades of research, however, treatment options remain limited. In ischemic stroke, the primary focus of treatment is reperfusion. Currently, the only drug approved for the treatment of ischemic stroke is recombinant tissue plasminogen activator (rtPA, alteplase), which has a limited time window for administration and increases the risk for subsequent hemorrhage. Consequently, only a small percentage of patients receive rtPA treatment [1]. While this treatment is effective in opening up occluded cerebral vessels in some patients and can lead to improved outcomes after ischemic stroke, there are currently no approved treatments for the myriad of damaging pathological processes that persist in the brain long after the acute stage. These include the processes of inflammation, excitotoxicity, oxidative stress, apoptosis, and edema resulting from disruption of the blood-brain barrier [2]. In hemorrhagic stroke, additional processes include physical damage from the mass of accumulated blood itself, cytotoxicity of blood components, and vasospasm in subarachnoid hemorrhage [3, 4].

A considerable amount of research has been invested into the development of novel treatments capable of protecting the brain from damage following stroke, with limited success. Numerous neuroprotective treatments have been identified that show great promise in animal models of stroke. Unfortunately, nearly all have failed to provide protection in human trials. The purpose of this review is to provide an overview of targets for neuroprotection in stroke and examples of current research on potential neuroprotective treatments. Several reviews of neuroprotection in both ischemic and hemorrhagic stroke have already been published in the last few years [5–9]. This paper will therefore concentrate only on the most recent research in this field. In addition, the primary focus will be on those treatments that have shown promise in animal models or human patients, as opposed to those that to date have only shown protection *in vitro* or in cell culture.

## 2. Animal Models of Stroke

A significant amount of research on neuroprotection in stroke is performed using animal models. A large variety of methods for inducing stroke in animals have been developed, and each is unique in its pathology and the effect of various neuroprotective agents. Since the results of experiments using a particular neuroprotective strategy may be dependent on the specific model used, it is necessary to understand these models and how they differ. The majority of stroke models use rodents and can be categorized by the type of stroke that they are designed to replicate. Models of ischemic stroke exhibit the greatest diversity in the types of procedures used; however most involve occlusion of one or more blood vessels. In focal ischemia models, typically only one vessel is occluded, the most common being middle cerebral artery occlusion (MCAO). Global ischemia models often involve bilateral occlusion of the common carotid arteries (CCAO) and may also include bilateral occlusion of another vessel such as the vertebral artery (4 vessel models). There are also 3 vessel occlusion models that combine bilateral common carotid occlusion with unilateral occlusion of another vessel. Models of ischemic stroke can also involve either permanent ischemia or transient ischemia with subsequent reperfusion. Models of hemorrhagic stroke typically involve the introduction of autologous blood into the brain by direct injection or procedures that cause rupture of a cerebral blood vessel. Other, less common stroke models exist but will not be discussed here. More detailed discussions of animal stroke models can be found in several reviews [10–13].

## 3. Targets for Neuroprotection in Stroke

### 3.1. Inflammation

**Figure 1:**
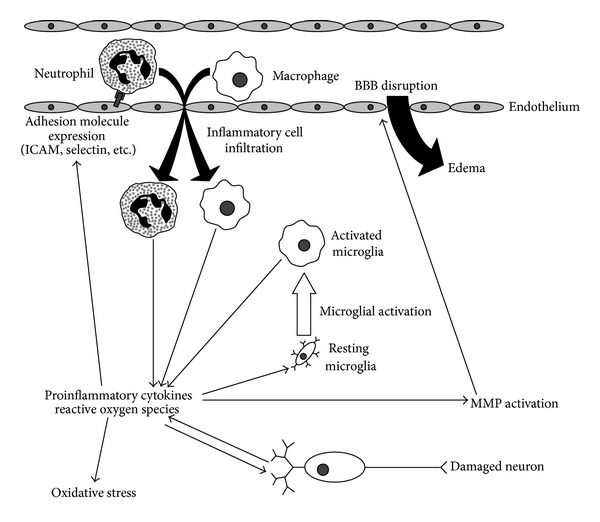
Damaging inflammatory mechanisms in stroke. Proinflammatory cytokines and reactive oxygen species released by damaged neurons lead to the activation of microglia and the expression of cellular adhesion molecules on endothelial cells and migrating inflammatory cells. Infiltrating inflammatory cells and activated microglia secrete additional cytokines and oxygen species, resulting in further tissue damage, oxidative stress, and activation of matrix metalloproteinases leading to disruption of the blood-brain barrier and edema.

A significant amount of the research being performed on neuroprotection following stroke is concentrated on mitigating the effects of inflammation. An overview of the inflammatory process in the brain after stroke is shown in Figure 1. Following ischemia and reperfusion, damaged brain tissue secretes cytokines and chemokines that recruit inflammatory cells to the injured area [14, 15]. These cells release their own secretory factors, which can build up to toxic levels. Inflammatory processes also result in the production of reactive oxygen species, leading to oxidative stress and activation of matrix metalloproteinases (MMPs), causing disruption of the blood-brain barrier (BBB) and edema. On the other hand, inflammation has beneficial effects as well, such as increasing blood flow to the affected area and the removal of damaged tissue by phagocytic cells and MMPs. The positive versus negative effects of inflammation following stroke and the appropriateness of intervention are a topic that is often debated [16]. It is generally considered, however, that inflammation does more harm than good after stroke, especially in the early stages. One important molecule resulting in cell damage and death following stroke is tumor necrosis factor alpha (TNF*α*). TNF*α* interacts with two receptors, R1 and R2, that mediate death signals via the Fas associated death domain (FADD) and inflammation via the nuclear factor kappa-light-chain enhancer of activated *B* cells (NF**κ**B), respectively [17]. Activation of the NF**κ**B pathway is commonly used as an indicator of inflammation in stroke studies. The interleukins are another important set of molecules in the process of inflammation. Interleukin-1 (IL-1) is proinflammatory, whereas IL-10 is anti-inflammatory and IL-6 has both pro- and anti-inflammatory effects [17]. Antagonists of the IL-1 receptor have been shown to be neuroprotective when administered at reperfusion in comorbid tMCAO rats [18].

As one of the early initiators of inflammation after stroke, TNF*α* is an excellent target for neuroprotective treatments. Perhaps the most straightforward way to block the effects of TNF*α* is to prevent or reduce its production. The thalidomide analog 3,6′-dithiothalidomide (3,6′-DT) is an inhibitor of TNF*α* synthesis that has been shown to reduce the number of activated inflammatory cells in the brain after ischemic stroke in mice, as well as the extent of BBB disruption [19]. Caffeic acid ester fraction reduces infarct volume and improves performance on behavioral tests in rats subjected to MCAO [20]. Further experiments with cultured microglia suggest that this effect is due to inhibition of the production of TNF*α*, as well as nitric oxide (NO) and IL-1*β*. Atorvastatin suppresses TNF*α* levels in a rat model of intracerebral hemorrhage, reducing brain water content and activation of microglia [21]. Another method of action against TNF*α* is the use of decoy receptors. Fusion proteins consisting of TNF receptor linked to a monoclonal antibody are capable of crossing the blood-brain barrier and significantly reduce infarct volumes after tMCAO in mice [22].

Activation of NF*κ*B by TNF*α* initiates a signaling cascade that regulates a number of inflammatory processes, making it a good point of intervention. Honokiol has been shown to suppress the activation of NF*κ*B in ischemic mice as well as levels of TNF*α* and significantly reduces brain water content [23]. Rosmarinic acid blocks activation of NF*κ*B by TNF*α* after tMCAO in diabetic rats and reduces edema and tissue damage [24]. Suppression of NF*κ*B activity by angiotensin-(1–7) reduces infarct volumes, improves neurological deficits, and decreases oxidative stress in rats subjected to pMCAO [25]. Kaempferol glycosides inhibit the activation of NF*κ*B as well as the signal transducer and activator of transcription 3 (STAT3), another proinflammatory transcription factor, in tMCAO rats, resulting in reduced infarct volume and neurological deficits [26]. It should be noted that not all NF*κ*B activity is harmful, and harmful activation of the NF*κ*B complex is associated with abnormal acetylation of the RelA subunit. Using a combination of an inhibitor of one type of deacetylase and an activator of another, it is possible to produce a RelA acetylation similar to that seen in the beneficial phenomenon known as ischemic preconditioning, resulting in neuroprotection in mice exposed to tMCAO [27].

The various signaling cascades induced by stroke lead to the activation and recruitment of inflammatory cells to the site of injury. In the early stages of stroke, prior to the infiltration of neutrophils and macrophages from other locations, resident microglia are the primary inflammatory cells in the brain. Microglia continue to be involved well into long term recovery and have been observed 28 days following stroke in MCAO rats [28]. Although microglia serve a beneficial purpose by removing dead tissue, they also release secretory factors that can accumulate to toxic levels, particularly in cases of excess activation such as stroke. Accordingly, treatments that limit microglial activation often have neuroprotective effects. The ginseng metabolite compound K suppresses microglial activation by inhibiting multiple upstream signaling molecules and is neuroprotective in MCAO mice [29]. Sesamin is neuroprotective in a mouse model of ICH and has been shown to prevent an increase in microglial cells by keeping them in their resting state [30]. Retinoids are also neuroprotective in models of ICH and reduce levels of activated microglia even with posttreatment [31]. Alternatively, increasing the reactivity of microglia can also have neuroprotective effects. The ATP-dependent potassium channel blocker glibenclamide increases the phagocytic capacity of microglia, resulting in improved neurological outcome, reduced infarct volume, and enhanced neurogenesis in rats subjected to transient or permanent MCAO [32–34]. Activation of the microglial alpha-7 nicotinic acetylcholine receptor induces expression of the heme oxygenase-1 (HO-1) gene, which is associated with neuroprotection in mice after photothrombotic stroke [35].

The processes involved in inflammation may not only directly contribute to brain damage following stroke but may also activate secondary mechanisms that lead to further damage. The activity of large numbers of inflammatory cells in the affected area, combined with low oxygen and ATP levels, leads to the formation of reactive oxygen species and the onset of oxidative stress. Activation of MMPs, while important for the removal of dead tissue and the ability of immune cells to enter the brain, may also result in disruption of the blood-brain barrier and edema due to an influx of water. These topics will be discussed separately in the following sections.

### 3.2. Oxidative Stress

The production of reactive oxygen species (ROS) and other free radicals during stroke is a consequence of not only inflammation but also excitotoxicity and the inhibition of cellular respiration in a low oxygen environment [36]. These molecules, such as hydroxyl radical, superoxide, and peroxynitrite, are highly reactive and damaging to multiple cellular components, leading to cell death. One way of reducing oxidative stress is to reduce the production of free radicals. Although nitric oxide is a normal signaling molecule in the body and has beneficial effects in stroke, larger amounts resulting from increased activity of the induced nitric oxide synthase (iNOS) can lead to aberrant signaling and or react with superoxide to produce peroxynitrite. Nebivolol decreases the expression of iNOS following bilateral CCAO in rats and increases expression of the beneficial endothelial nitric oxide synthase (eNOS), leading to a reduction in histopathological changes [37]. Another source of ROS is the nicotinamide adenine dinucleotide phosphate (NADPH) oxidases, and inhibitors of these enzymes could be beneficial in stroke [38]. Another method of protection is to induce endogenous mechanisms for the removal of free radicals in the body. Hydrogen sulfide gas increases the activity of superoxide dismutase and glutathione peroxidase in rats subjected to focal cerebral ischemia, resulting in decreased injury to neuronal mitochondria and a subsequent reduction in markers of apoptosis [39]. Hydrogen-rich saline increases endogenous antioxidant enzyme activity and decreases the amount of oxidative products in pMCAO rats [40]. A nucleic acid-based product improves the antioxidant status of neuronal mitochondria after transient ischemia in rats [41]. Alternatively, exogenous compounds with free radical scavenging properties can be used. The novel compound MnTm4PyP mimics the activity of endogenous manganese superoxide dismutase, and reduces infarct volume and neurological deficit after MCAO in mice [42]. An extract of *Ocimum sanctum* protects tMCAO rats by preserving reduced glutathione content and antioxidant enzyme activity [43]. Hydrogen gas has been shown to neutralize free radicals and has beneficial effects on several contributors to early brain injury after subarachnoid hemorrhage in rats [44]. Furthermore, noneffective doses of the free radical scavenger lipoic acid enhance the neuroprotective effects of the NADPH oxidase inhibitor apocynin when given in combination to tMCAO rats [45]. Inhibition of downstream signaling pathways leading to oxidative stress-induced damage, rather than the direct removal of ROS, may also have beneficial effects. The antioxidant N-tert-butyl-*α*-phenylnitrone suppresses expression of complement component 3, a mediator of inflammation that is induced by oxidative stress, in mice after transient focal cerebral ischemia [46].

### 3.3. Blood-Brain Barrier Disruption

Disruption of the blood-brain barrier following stroke is commonly associated with the action of two matrix metalloproteinases, MMP-2 and MMP-9. MMP-2 is constitutively expressed at low levels in normal brain tissue; however stroke increases its expression and activity and also induces the expression of MMP-9. MMP-2 cleaves and activates MMP-9, which degrades components of the basement membrane in vascular walls and leads to BBB disruption. Other factors involved in BBB permeability after stroke include the extent of tight junction formation between endothelial cells and the effects of treatment with tissue plasminogen activator. The activity of MMPs is regulated endogenously by the tissue inhibitor of matrix metalloproteinase (TIMP), and treatments that stimulate it may be of significant benefit in protection against stroke-related brain damage. Inhibition of the expression and activity of MMPs by other means may be protective as well. Ethanol has been shown to inhibit the increase in MMP-2 and MMP-9 expression after tMCAO in rats and significantly reduces brain edema [47]. *Apocynum venetum* leaf extract (AVLE) also alleviates symptoms of BBB disruption in tMCAO rats by inhibiting the expression and activity of MMPs [48]. Hyperbaric oxygen treatment improved BBB function in a rat embolic stroke model through modulation of MMP-9 but displayed reduced effectiveness when administered in combination with tPA [49]. It may therefore be of limited benefit in those stroke patients who receive tPA. Treatments that increase the formation of tight junctions may also provide neuroprotection following stroke. Doxycycline increases the expression of tight junction proteins in tMCAO rats and also inhibits MMPs [50]. Kruppel-like factor 2 (KLF2) protects against tMCAO in mice by regulation of the tight junction component occluding [51]. The c-Jun N-terminal kinase (JNK) inhibitor SP600125 restores vascular tight junctions and alleviates BBB disruption in a rat model of subarachnoid hemorrhage [52]. The GTPase RhoA is known to play an important role in the regulation of endothelial tight junctions, and inhibition of RhoA by fibroblast growth factor preserves BBB integrity in a mouse model of intracerebral hemorrhage [53]. Additional neuroprotective strategies include those that alleviate the negative effects of tPA. High density lipoproteins have been shown to reduce hemorrhagic transformation and improve BBB integrity following tPA treatment in experimental models [54]. Neurotrophic factors can also be used to stimulate repair mechanisms and restore BBB function. Pigment epithelium-derived factor (PEDF), for example, has been shown to have beneficial effects on both BBB permeability and lesion volume after ischemia-reperfusion in rats [55].

### 3.4. Excitotoxicity

During stroke, depletion of neuronal oxygen and energy reserves leads to the release of toxic amounts of the neurotransmitter glutamate into the extracellular space [2, 17]. The subsequent activation of glutamate receptors causes an influx of calcium and neuronal depolarization, resulting in aberrant activation of numerous calcium-dependent pathways in the brain and initiation of the processes of necrosis, apoptosis, and autophagy. Glutamate excitotoxicity therefore plays a significant role in the pathology of stroke, and a large number of research studies are devoted to suppressing its effects. One method of neuroprotection against excitotoxicity is to reduce the amount of glutamate release during stroke. The *Ginkgo biloba* extract EGb761 has been shown to significantly decrease striatal glutamate levels in mice subjected to MCAO, accompanied by reduced neurodegeneration and edema [56]. The individual constituents of EGb761 also have neuroprotective properties [57]. Another method of protection is to block the action of glutamate receptors in the brain. The microRNA miR-223 reduces expression of the glutamate receptor subunits GluR2 and NR2B, and is neuroprotective against transient global ischemia [58]. Activation of the transient receptor potential vanilloid 4 (TRPV4) is associated with increased activity of the NMDA receptor. HC-067047, an antagonist of TRPV4, reduces the extent of infarct after tMCAO in mice [59]. Magnesium sulfate is an antagonist of the N-methyl-D-aspartate (NMDA) subtype of glutamate receptor and in some studies has been shown to improve recovery in humans with acute ischemic stroke [60]. On the other hand, large scale clinical trials such as the intravenous magnesium efficacy in stroke (IMAGES) trial concluded that magnesium did not significantly improve outcome in ischemic stroke patients but may be beneficial in lacunar stroke [61]. It is possible that the timing of treatment is critical; therefore the field administration of stroke therapy-magnesium (FAST-MAG) trial was devised to test the benefits of magnesium given prior to arrival at a hospital. Results from a pilot trial suggest that early administration of magnesium may have benefits, and phase III trials are currently underway [62]. Inhibiting the influx of calcium into cells following glutamate receptor activation can also be beneficial. Overexpression of the transient receptor potential canonical 6 (TRPC6) suppresses the increase in calcium induced by NMDA and reduces infarct size and mortality in mice [63]. Hyperforin, an activator of TRPC6, has also been found to be neuroprotective following tMCAO in rats [64]. The compound ginsenoside-Rd decreases expression of the calcium channel TRPM7 in MCAO rats, which may be partially responsible for its neuroprotective effects [65].

### 3.5. Apoptosis

**Figure 2:**
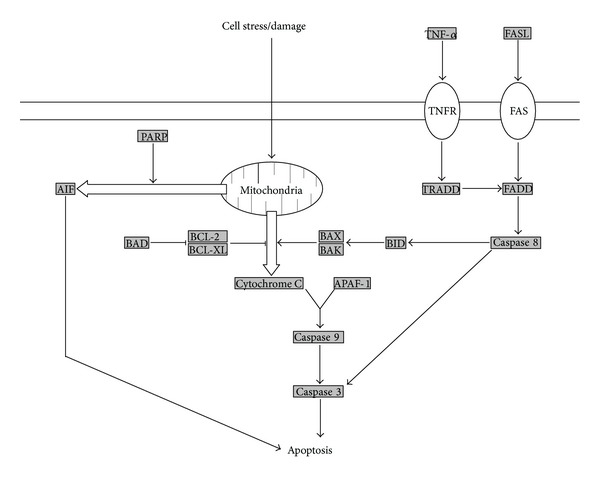
Mechanisms of induction of apoptosis. In the classical pathway, mitochondria release cytochrome C in response to cell stress and damage, leading to activation of caspase 9 and subsequent activation of caspase 3 and other effectors of apoptosis. Alternatively, mitochondria may also release apoptosis-inducing factor (AIF), which leads to apoptosis by a caspase independent mechanism. The death receptor pathway involves the activation of FADD by various cell signal receptors, followed by activation of caspase 8 and the subsequent caspase cascade leading to apoptosis.

During stroke, the diminished supply of oxygen and glucose to the brain leads to reduced cellular metabolism and depletion of energy stores. Combined with tissue damage due to mechanisms such as those mentioned above, cell death by either necrosis or apoptosis may be initiated. In the context of intervention, apoptosis is preferable to necrosis because it can be blocked by various treatments, allowing damaged tissue to be rescued. Cells within the core infarct typically die by necrosis, whereas those in the penumbra die by apoptosis. The primary factor in determining which mechanism of cell death occurs is the level of ATP within the cell [66]. ATP is required for the process of apoptosis, and cells with insufficient ATP stores will die by necrosis instead. Apoptosis can occur by several pathways, as shown in Figure 2. The mitochondrial pathway can proceed through either caspase dependent or caspase independent mechanisms. Alternatively, apoptosis may be induced by the death receptor pathway.

In the caspase dependent pathway of mitochondrial apoptosis, release of cytochrome C from mitochondria results in activation of caspase 3, which initiates a caspase cascade leading to the degradation of cellular components and cell death. Caspase 3 activity is commonly used as an indicator of apoptosis. Reduction of activated caspase 3 levels is therefore a goal of many neuroprotective treatments. Tanshinone IIA decreases the levels of cleaved caspase 3 in tMCAO rats, resulting in a reduction in infarct volume, edema, and neurological deficits. Diallyl sulfide reduces expression of caspase 3 and increases expression of BCL-2, an endogenous antiapoptotic protein, in tMCAO rats as well. Hypothermia has been shown to reduce caspase 3 levels for up to 1 week after focal cerebral ischemia in rats [67]. Pioglitazone, an agonist of the peroxisome proliferator-activated receptor *γ* (PPAR*γ*), activates STAT3 in tMCAO rats, leading to changes in the expression of antiapoptotic genes and reduced levels of caspase 3 [68]. Caspase 3 is not the only potential therapeutic target within this pathway, however. The cellular inhibitors of apoptosis (cIAPs) are endogenous molecules that bind to caspases and block their activation. Ischemic preconditioning has been shown to increase the levels of cIAP1 in neurons and reduce apoptosis following unilateral CCAO in rats [69].

Although apoptosis is most commonly associated with the caspase dependent mitochondrial pathway, other pathways also contribute to cell death after stroke, and neuroprotective agents that act upon these pathways are being investigated. The caspase independent pathway of apoptosis is characterized by mitochondrial release of apoptosis-inducing factor (AIF), which is stimulated by the activity of poly(ADP-ribose) polymerase (PARP). Several treatments that inhibit the caspase dependent pathway of apoptosis have also been shown to inhibit the caspase independent pathway as well. Ethanol administration decreases the expression of both caspase 3 and AIF up to 24 hours after tMCAO in rats [70]. The nitric oxide donor (S)-ZJM-289 suppresses the release of both cytochrome C and AIF from mitochondria and significantly reduces injury in MCAO rats [71]. Cyclosporin A has been shown to decrease the expression of caspase 3, AIF, and cytochrome C in a rat model of SAH [72]. Other compounds have been identified that target the caspase independent pathway specifically. Ginsenoside-Rd has been shown to inhibit PARP-1 activity and AIF release in rats subjected to MCAO [73]. The death receptor pathway of apoptosis differs from the other two in that mitochondria are not required for its induction. Similar to the caspase dependent pathway of mitochondrial apoptosis, however, the death receptor pathway also uses caspase 3 as an effector. Treatments that affect the level and activity of caspase 3 may therefore block this pathway as well. Treatments that target this pathway directly have also been identified. Muscone decreases the expression of the death receptor FAS and reduces apoptosis in MCAO rats [74]. Antibodies against TNF*α*, another initiator of the death receptor pathway, block changes in the expression of caspase 3 after SAH in rats [75].

### 3.6. Autophagy

**Figure 3:**
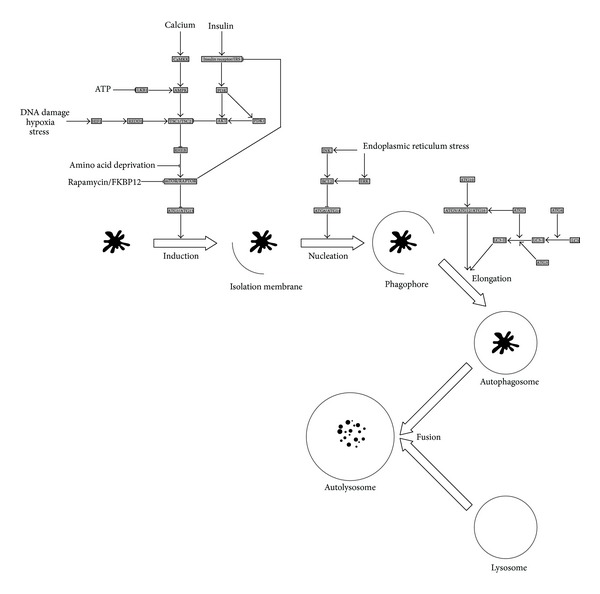
The process of autophagy and its regulation. Induction of autophagy is inhibited by mTOR, the activity of which is controlled by numerous upstream signaling pathways that respond to metabolic activity, energy status, and tissue damage. Progression of autophagy requires several members of the ATG protein family, leading to the production of a membranous structure that engulfs damaged cellular components to form the autophagosome. Subsequent fusion of the autophagosome with a lysosome results in degradation of the damaged components. Proteins involved in the regulation of autophagy are shown in shaded boxes. Positive interactions are denoted by arrows and negative interactions by lines with flat ends.

The role of autophagy in stroke has only recently begun to be elucidated and is not fully understood. Autophagy appears to have a dual role in the response to cellular damage, absorbing damaged components as a protective measure in some cells and serving as a mechanism of cell death in others. Autophagy is mediated by the ATG family of proteins and regulated by a number of nutrient and energy sensing pathways that converge on the mammalian target of rapamycin (mTOR) [76, 77]. An overview of the process of autophagy and its regulation is shown in Figure 3. Induction of autophagy prevents cell death by apoptosis and in this respect is considered to be beneficial. The drug rapamycin is an inhibitor of mTOR that leads to induction of autophagy. In a rat model of subarachnoid hemorrhage, rapamycin suppressed apoptosis and reduced neurological deficits, whereas inhibition of autophagy increased the amount of brain damage [78]. Activity of the tuberous sclerosis complex (TSC) also leads to mTOR inhibition and induction of autophagy. Suppression of the TSC1 subunit of this complex has been shown to increase the vulnerability of hippocampal neurons to cell death after ischemia *in vivo* [79]. Alternatively, the inhibition of autophagy can also be neuroprotective. In pMCAO rats, ischemic postconditioning inhibited the induction of autophagy and reduced infarct size and edema [80]. A chemical inhibitor of autophagy had similar effects, whereas rapamycin partially reversed them. It is hopeful that further research will determine if the overall effect of autophagy in stroke is beneficial or harmful.

## 4. Ischemic Tolerance and the Effects of Pre- and Postconditioning

The brain is highly susceptible to ischemia, and numerous endogenous mechanisms exist to protect neural tissue from its effects. Although these mechanisms are naturally stimulated in response to stroke, they may also be artificially induced by nondamaging techniques to produce a protective state known as ischemic tolerance [81, 82]. Preconditioning is a neuroprotective strategy by which ischemic tolerance is induced prior to stroke in order to protect the brain from subsequent damage and potentially could be used as a preventative measure in high risk individuals or as a precaution against secondary stroke following medical procedures such as aneurysm repair or cardiac surgery. In the case of naturally occurring strokes which cannot be predicted, postconditioning may be a therapeutic strategy that could be used afterward to accelerate or enhance protective mechanisms or as a precaution against stroke recurrence. Preconditioning and ischemic tolerance have already been extensively reviewed and will only be covered briefly here [83–86]. Some evidence suggests, however, that, although preconditioning may be beneficial in the short term, long term structural changes in the brain indicate that tissue damage is merely being postponed [87]. Clinical studies are needed to test the safety and efficacy of these novel strategies in humans.

Ischemic tolerance can be induced directly by many conditioning mechanisms, including hypoxia/hyperoxia, hypothermia/hyperthermia, drug treatments, blood vessel occlusion, and dietary restriction [81]. Ischemic preconditioning has been shown to downregulate components of metabolic pathways, such as adenosine 5′-monophosphate-activated protein kinase (AMPK) [88]. Preconditioning by the anesthetics sevoflurane and isoflurane inhibits the expression of proapoptotic genes and upregulates antiapoptotic genes in MCAO rats [89]. Potential mediators of preconditioning include the lymphocyte cell kinase (LCK) and sodium/calcium exchangers (NCX) [90–92]. Postconditioning inhibits MMP9 expression and subsequent degradation of the extracellular matrix in MCAO rats [93]. Ischemic tolerance in the brain can also be stimulated remotely, for example, by application of a tourniquet to one of the limbs. Remote preconditioning is protective in a mouse embolic stroke model, both by itself and in combination with tissue plasminogen activator [94]. It also provides protection by upregulation of eNOS in rats subjected to global ischemia and reperfusion [95]. Remote preconditioning has even been shown to have beneficial effects in human patients with subarachnoid hemorrhage, but further studies are needed [96].

## 5. Neuroprotection in Hemorrhagic Stroke

The pathologies of ischemic and hemorrhagic stroke share many of the same damaging processes, such as inflammation, oxidative stress, and excitotoxicity. Treatments that are neuroprotective in one may also be beneficial in the other, and several examples of protection in models of hemorrhagic stroke have already been given in the previous sections. Processes such as cytotoxicity, however, are unique to hemorrhagic stroke and are directly related to the accumulation of blood in the brain. Multiple blood components are believed to contribute to tissue damage after hemorrhage, particularly hemoglobin. Haptoglobin and hemopexin are endogenous proteins that bind and remove hemoglobin and its degradation products, respectively, and enhancement of their activity may be protective [3]. Heme oxygenase (HO) is another endogenous protein that converts heme to iron and other products. Free iron reacts with hydrogen peroxide to form hydroxyl radicals, leading to oxidative stress. Inhibition of HO or chelation of iron by agents such as deferoxamine could have beneficial effects. Valproic acid decreases the expression of HO-1 following ICH in rats and attenuated cell death [97]. Combination treatment of deferoxamine and statins has been associated with improved results in behavioral tests in rats after ICH [98]. In addition to cytotoxicity, cerebral vasospasm is a significant contributor to death and disability after subarachnoid hemorrhage. Because of its serious nature, a considerable amount of research is dedicated to mitigating its effects. Hydrogen-rich saline has been shown to decrease vasospasm in rats after experimental SAH, possibly by suppressing the effects of inflammation and oxidative stress [99]. Vasospasm in SAH rats is also reduced by an analog of *α*-melanocyte stimulating hormone and is accompanied by changes in the expression of factors involved in tissue damage and repair [100].

## 6. Benefits of Combination Therapy for Neuroprotection in Stroke

The pathology of stroke is complex, with multiple overlapping processes leading to either damage or protection. Although considerable neuroprotection can be gained by targeting just one of these processes, the potential benefit is even greater if multiple mechanisms of damage can be suppressed at the same time. Targeting the same pathway with more than one neuroprotective agent can also be beneficial. Consequently, combination therapy with more than one drug has proven to be effective in several experimental studies. The additional benefit may be small, additive, or even synergistic in some cases. Progesterone plus vitamin D hormone, for example, reduces infarct size and neurological deficits in tMCAO rats to a greater extent than either treatment alone [101]. The combination of the anti-inflammatory properties of atorvastatin and the antioxidant properties of probucol also produces increased neuroprotection in pMCAO rats [102]. Simvastatin combined with granulocyte colony-stimulating factor (G-CSF) reduced recovery time in a rat model of intracerebral hemorrhage and improved outcome [103]. Another strategy for combination therapy is the use of one drug to block the negative effects of another, such as tPA. Mild hypothermia, high-density lipoproteins, activated protein C analog, and fingolimod have all been shown to reduce the incidence of hemorrhagic transformation following administration of tPA in several animal models [54, 104–106].

## 7. Neuroprotective Treatments with Pleiotropic Effects

Although combination therapy can produce additional benefits in some cases, not all treatments are compatible with each other. Some combinations may have antagonistic effects, producing less benefit than either treatment alone. In some cases, combination therapies may enhance damage. Furthermore, combination treatment requires significantly more testing to determine safety and effectiveness than a single compound. There is therefore considerable interest in the study of treatments with beneficial effects on more than one mechanism of stroke-related damage. Some of these treatments have only recently been discovered (Table 1) and are still in the early stages of investigation. Others, however, have already been the subject of considerable study and show great promise for the treatment of stroke, as summarized below.

### 7.1. Minocycline

Minocycline is a broad spectrum antibiotic which, in addition to its antibacterial activity, also has anti-inflammatory and antiapoptotic effects [107]. Consequently, it has been shown to be protective in a number of diseases including stroke. Minocycline is one of the few neuroprotective agents in animal studies that has also been proven effective in human trials. In one recent trial, oral administration of minocycline resulted in significantly improved outcomes as long as three months after stroke [108]. Furthermore, animal studies indicate that additional benefits are to be gained by combining minocycline with other neuroprotective strategies. Minocycline reduces the risk of subsequent hemorrhage following administration of tissue plasminogen activator in diabetic rats subjected to focal embolic stroke [109]. It also reduces infarct size and suppresses several hallmarks of inflammation. Minocycline plus normobaric hyperoxia has a synergistic effect on the reduction in infarct volume following tMCAO in rats and has a positive effect on hemispheric swelling that was not seen with either treatment alone [110]. This combination also resulted in greater inhibition of apoptosis and MMP activation. Minocycline improves recovery after transplantation of bone marrow mononuclear cells into ischemic rats, presumably by inhibition of microglial activation [111, 112]. It also preconditions neural stem cells against oxidative stress, producing reduced infarct size and improved neurological performance following transplantation in rats exposed to tMCAO [113].

### 7.2. Carnosine

Carnosine is a naturally occurring dipeptide that has both antioxidant and antiexcitotoxic properties [114, 115]. It also is an efficient chelator of metal ions such as zinc, which is required for the activity of matrix metalloproteinases. Preclinical studies have shown that carnosine is well tolerated and produces robust neuroprotection in animal models of both transient and permanent ischemia [116]. It also readily crosses the intact blood-brain barrier, which allows it to be administered even in the early stages of stroke. Carnosine reduces glutamate excitotoxicity in pMCAO mice, resulting in reduced infarct size and improved neurologic function [117]. In another study, carnosine was shown to decrease infarct size, MMP activity, and levels of reactive oxygen species [118]. Carnosine can also be cleaved by carnosinase into the amino acids alanine and histidine, which are neuroprotective as well. Bestatin, an inhibitor of carnosinase, increases damage in pMCAO mice [119].

### 7.3. Asiatic Acid

Asiatic acid is a plant-derived compound with effects on oxidative stress, inflammation, and excitotoxicity that has been shown to be beneficial in the treatment of wound healing, beta-amyloid toxicity, and liver injury. Recent evidence suggests that it may be neuroprotective in stroke as well. In pMCAO mice, asiatic acid reduces infarct size and improves neurologic scores, possibly by suppression of mitochondrial damage and BBB disruption [120]. Subsequently, asiatic acid was also found to be neuroprotective in multiple models of ischemia in rats by inhibiting mitochondrial damage and MMP-9 activation [121]. Asiatic acid also blocks the negative effects of excitotoxicity in mice following exposure to glutamate [122]. An extract of *Centella asiatica* has been shown to improve several markers of behavioral function and improved the antioxidant status in tMCAO rats [123]. This extract contains asiatic acid as well as several other related compounds that also have neuroprotective properties. Further investigation of this class of molecules is therefore warranted to determine the extent of their effects.

### 7.4. Hypothermia

The beneficial effects of cooling the body after injury are well known, and induction of hypothermia is currently used in clinical practice to prevent secondary brain injury after cardiac arrest and resuscitation, perinatal or neonatal asphyxia, and head trauma [124–126]. Considerable evidence suggests that hypothermia may be beneficial in the treatment of stroke as well. Microarray experiments have determined that neuroprotection resulting from hypothermia in MCAO rats is associated with changes in the expression of genes involved in the processes of inflammation, apoptosis, and calcium regulation [127]. Hypothermia induction by the neurotensin receptor 1 (NTR1) agonist ABS-201 reduces infarct volume and suppresses cell death by apoptosis and autophagy in mice subjected to focal ischemia [128]. A recent meta-analysis of human clinical trials, on the other hand, found no benefit of hypothermia in stroke patients [129]. These trials had a limited number of subjects, however, and larger trials are currently in progress, for example, EuroHYP-1 and ICTuS2/3 [130].

### 7.5. Flavonoids

In some cases, whole classes of molecules are known for having multiple neuroprotective effects. One such group of compounds that are currently the subject of intensive research are the flavonoids. These molecules are naturally occurring compounds that readily cross the blood-brain barrier and are well known for their protective effects. The flavonoids in cocoa, for example, have antioxidant properties and also promote perfusion, angiogenesis, and neurogenesis in the brain [141]. Xanthohumol has been found to have anti-inflammatory, anti-apoptotic, antioxidant, and antithrombotic properties. Following MCAO in rats, xanthohumol decreases the levels of TNF*α*, hypoxia-inducible factor 1 alpha (HIF-1*α*), and inducible nitric oxide synthase (iNOS) [142]. It also reduces expression of activated caspase 3, scavenges hydroxyl radicals, and inhibits platelet aggregation. Treatment with naringenin results in neuroprotection in tMCAO rats through both antioxidant and anti-inflammatory mechanisms [143]. Galangin improves cerebral blood blow, inhibits apoptosis, and protects mitochondrial function after MCAO in rats [144]. In tMCAO mice, fisetin protects the brain against ischemic injury by suppressing activation of cerebral inflammatory cells and inhibiting the migration of macrophages and dendritic cells into the brain [145]. In models of intracerebral hemorrhage, baicalin has been found to attenuate edema of the brain and inhibit apoptosis [146].

### 7.6. Cannabinoids

Another major class of molecules with multiple beneficial effects in stroke are the cannabinoids. These compounds are primarily known for their anti-inflammatory effects in many diseases including stroke [147]. Recently, however, evidence suggests that cannabinoids may also have antioxidant and antiapoptotic effects [148]. The cannabinoid receptor agonists WIN55,212-2 and JWH-133 reduce activation of microglia and macrophages after induction of ischemia in mice and rats, resulting in reduced infarct size and neurological impairment, as well as protection of oligodendrocyte precursor cells [149–151]. Another receptor agonist, TAK-937, provides neuroprotection in tMCAO rats, and the neuroprotective effect is increased when given in combination with hypothermia [152]. Furthermore, TAK-937 not only is effective in rodent models of stroke but also has been shown to reduce infarct volume in nonhuman primates [153]. This specific compound is therefore of considerable interest for future use in human trials.

## 8. Neuroprotective Agents in Human Clinical Stroke Trials

Perhaps the greatest challenge in the study of neuroprotection in stroke is the translation of animal studies to humans. Numerous treatments that produce robust protection in rodents have failed to provide significant benefit in clinical trials. The various theories on the reason for this failure have already been discussed elsewhere and will not be covered here [5, 154, 155]. Amid the abundance of discouraging results, however, a small number of neuroprotective strategies have shown promise in human stroke patients. A brief summary of recently completed clinical trials for the study of neuroprotection in ischemic stroke and subarachnoid hemorrhage is provided below. A discussion of recent clinical trials for neuroprotection in intracerebral hemorrhage is already available [8].

### 8.1. International Citicoline Trial on Acute Stroke (ICTUS)

Citicoline is a nutritional supplement that not only is commonly used to improve memory retention but also has been shown to prevent neuronal degeneration and improve visual function. It has already been approved in some countries for the treatment of acute ischemic stroke. A randomized, placebo controlled trial was conducted to evaluate the efficacy of citicoline in patients with moderate to severe acute ischemic stroke [156]. A total of 2298 patients were administered either citicoline (1000 mg every 12 hours) or placebo for up to 6 weeks. Outcome was determined at 90 days based on the National Institute of Health Stroke Scale (NIHSS), modified Rankin Scale (mRS), and modified Barthel Index (mBI scores), plus the occurrence of intracranial hemorrhage, neurologic deterioration, or death. No significant difference in recovery was observed between the citicoline and placebo treatment groups.

### 8.2. Minocycline

Minocycline is an oral antibiotic with proven safety over years of use. In addition to its antibiotic properties, minocycline also has anti-inflammatory and antiapoptotic effects that have been shown to be neuroprotective in animal models of stroke and in previous human trials. The efficacy of oral minocycline was examined in a recent single-blinded open-label study [108]. Fifty patients with acute ischemic stroke were given either minocycline (200 mg/day) or placebo for five days and assessed for various indicators of outcome at 1, 7, 30, and 90 days. Patients receiving minocycline showed significant improvement after 30 days in NIHSS, mBI, and mRS scores. NIHSS scores continued to be significantly improved at 90 days. Larger phase II and phase III trials are awaited.

### 8.3. Cerebrolysin

Cerebrolysin is a mixture of peptide fragments that mimics the action of neurotrophic factors and has been shown to be neuroprotective in a number of conditions such as hyperthermia-induced neurotoxicity, vascular dementia, Alzheimer's disease, traumatic brain injury, and stroke. A large, double-blind clinical trial was conducted to test the efficacy and safety of Cerebrolysin in patients with acute ischemic stroke [157]. A total of 1070 patients were administered aspirin and either Cerebrolysin (30 mL/day) or placebo over a period of 10 days. Although no significant difference between treatment groups was seen after 90 days, a positive trend was seen in those patients with an NIHSS score greater than 12.

### 8.4. Ginsenoside-Rd

Ginsenoside-Rd is a calcium channel antagonist that has been previously shown to be neuroprotective in human trials. An extended trial of ginsenoside-Rd was performed in 390 patients with acute ischemic stroke [158]. Subjects were administered ginsenoside-Rd or placebo intravenously over a 14-day period and evaluated using NIHSS and mRS scores for 90 days. Significant improvement was seen with ginsenoside-Rd in NIHSS scores at 15 days and mRS scores at 90 days.

### 8.5. Granulocyte-Colony Stimulating Factor (G-CSF)

G-CSF is a growth factor that stimulates the production and release of hematopoietic stem cells in bone marrow and may be beneficial in the enhancement of recovery after stroke. The safety of G-CSF was examined in a randomized phase IIb trial [159]. A total of 60 patients with either ischemic or hemorrhagic stroke were given G-CSF (10 *μ*g/kg) or placebo over 5 days and monitored for the frequency of adverse effects. G-CSF significantly increased white cell counts and produced a trend toward reduced infarct volume. No difference was seen between treatment groups in the frequency of adverse effects.

### 8.6. Evaluating Neuroprotection in Aneurysm Coiling Therapy (ENACT) Trial

The compound known as NA-1 (Tat-NR2B9c) is an inhibitor of the postsynaptic density-95 (PSD-95) protein that is neuroprotective in primate models of stroke. PSD-95 associates with the NMDA glutamate receptor subtype and contributes to the process of excitotoxicity, which is suppressed by treatment with NA-1. The safety and efficacy of NA-1 after endovascular aneurysm repair were examined in patients with iatrogenic stroke [160]. A total of 185 patients were treated for a ruptured or unruptured intracranial aneurysm by endovascular coiling, followed by administration of NA-1 or placebo. Evaluative criteria included the number and size of ischemic strokes occurring after the endovascular procedure, as well as adverse effects associated with NA-1 treatment. NA-1 was found to decrease the number, but not the size, of strokes occurring after surgery. No serious adverse effects of NA-1 treatment were observed.

### 8.7. Magnesium for Aneurysmal Subarachnoid Haemorrhage (MASH-2) Trial

Magnesium is a glutamate receptor antagonist that has been shown to be neuroprotective due to reduction of excitotoxicity following stroke. A multicenter phase III trial was conducted to determine the effect of magnesium therapy on outcome in aneurysmal subarachnoid hemorrhage [161]. A total of 1204 patients were administered intravenous magnesium sulfate (64 mmol/day) or placebo and evaluated for outcome on the modified Rankin Scale for up to 90 days. No improvement in outcome was seen with magnesium treatment versus controls. In addition, a retrospective analysis of 2047 patients from previous trials was also performed and similarly concluded that magnesium has no benefit in the treatment of aneurysmal subarachnoid hemorrhage.

### 8.8. Albumin in Subarachnoid Hemorrhage (ALISAH) Trial

Albumin is an endogenous protein that has been shown to have multiple neuroprotective effects, including antioxidant activity, inhibition of apoptosis, improved cellular metabolism, and reduced edema. A multicenter pilot trial was conducted to evaluate the safety and tolerability of human albumin in patients with subarachnoid hemorrhage [162]. A total of 47 patients were tested with three different dosage levels of human albumin, administered daily for 7 days. Albumin treatment was found to be well tolerated with minimal adverse effects at doses of 625 mg/kg and 1250 mg/kg, but not at higher levels. In addition, 1250 mg/kg albumin trended toward better outcome than the lower dosage level. A phase III trial to determine the efficacy of albumin in subarachnoid hemorrhage is currently in progress.

## 9. Summary

The pathology of stroke is incredibly complex, and treatment of its devastating effects is a continuing medical challenge. The topic of neuroprotection in stroke is equally complex, as can be seen by the wide variety of approaches currently being studied by the scientific community. On the one hand, no treatment or combination of treatments can be expected to encompass the entirety of damaging processes that occur during stroke. In this respect, the search for better therapies is never ending. On other hand, the availability of a large number of neuroprotective strategies increases the probability that one or more will ultimately prove to be effective. This fact is particularly relevant considering that the large majority of neuroprotective treatments developed in animal models have failed to produce significant benefits in human trials. As a result, treatment options for stroke are still limited. A few neuroprotective agents have shown promise, however, and it is hopeful that they may be approved for general use in the future.

The failure of preclinical studies to translate into clinical trials highlights the importance that these studies be properly designed. To this end, the Stroke Therapy Academic Industry Roundtable (STAIR) has developed a set of recommendations for the preclinical assessment of neuroprotective treatments [163]. These include consideration of the proper animal model, dosage level, and time points to be used, as well as the use of physiological monitoring and more than one measure of outcome. Recommendations for phase I/II clinical trials of potential stroke therapies have also been developed to facilitate the transition to phase III trials [164]. It is critical that both preclinical studies and clinical trials be designed to complement one another, in order to ensure that the results are comparable and to allow subsequent investigation of the reasons behind the success or failure of neuroprotective treatments in humans. It has also been proposed that, rather than proceeding directly from animal studies to clinical trials, international multicenter preclinical studies should be performed on promising neuroprotective agents to identify potential problems in translating from animals to humans [165, 166].

One complicating factor in the development of neuroprotective strategies is the dual nature of many of the processes that occur in the brain during stroke. The activity of MMPs, microglia, and other inflammatory cells, for example, can be either damaging or protective depending on the magnitude, location, and timing of their effects. Even mechanisms of cell death such as apoptosis and autophagy can be beneficial in the right circumstances. The development of potential neuroprotective treatments, therefore, must take both the positive and negative aspects of the stroke response into consideration, to ensure that they are administered under the conditions that are most appropriate and that will produce the greatest benefit.

## Figures and Tables

**Table 1:** Neuroprotective treatments with multiple beneficial effects in stroke.

Treatment	Species	Model	Benefits	Reference
ITH33/IQM9.21	Mouse	Ischemia	Antiexcitotoxic, antioxidant	Lorrio et al. 2013 [131]
TAT-M9	Mouse	Ischemia	Antioxidant, antiapoptotic	Guo et al. 2013 [132]
Glycyrrhizic acid	Rat	Ischemia	Anti-inflammatory, antiexcitotoxic, and antioxidant	Kim et al. 2012 [133]
Vitis amurensis extract	Rat	Ischemia	Anti-inflammatory, antiexcitotoxic, antioxidant, and antiapoptotic	Kim et al. 2012 [134]
*cis*-Hinokiresinols	Rat	Ischemia	Anti-inflammatory, antioxidant	Ju et al. 2013 [135]
Berberine	Rat	Ischemia	Anti-inflammatory, antiapoptotic	Zhang et al. 2012 [136]
S-Nitrosoglutathione	Rat	Ischemia	Antioxidant, BBB integrity	Khan et al. 2012 [137]
Taurine	Rat	Ischemia	Antiexcitotoxic, antioxidant, and antiapoptotic	Gharibani et al. 2013 [138]
MFG-E8	Rat	Ischemia	Anti-inflammatory, antiapoptotic	Cheyuo et al. 2012 [139]
Nitrone derivatives	Rat	Ischemia	Antiexcitotoxic, antioxidant	Sun et al. 2012 [140]
